# The ubiquitin ligase Peli1 inhibits ICOS and thereby Tfh-mediated immunity

**DOI:** 10.1038/s41423-021-00660-5

**Published:** 2021-03-11

**Authors:** Xinfang Huang, Shumeng Hao, Junli Liu, Yuanyuan Huang, Manman Liu, Chunyuan Xiao, Yan Wang, Siyu Pei, Tao Yu, Jing Xu, Haikun Wang, Dongfang Dai, Xiao Su, Yichuan Xiao

**Affiliations:** 1 Department of Rheumatology, Shanghai East Hospital, Tongji University, School of Medicine, Shanghai, China; 2 CAS Key Laboratory of Tissue Microenvironment and Tumor, Shanghai Institute of Nutrition and Health, Chinese Academy of Sciences, University of Chinese Academy of Sciences, Shanghai, China; 3 CAS Key Laboratory of Molecular Virology & Immunology, Unit of Respiratory Infection and Immunity, Institut Pasteur of Shanghai, Chinese Academy of Sciences, University of Chinese Academy of Sciences, Shanghai, China; 4 Department of Radiation Oncology, The Affiliated Cancer Hospital of Nanjing Medical University (Jiangsu Cancer Hospital) and Jiangsu Institute of Cancer Research, Nanjing, Jiangsu China

**Keywords:** T follicular helper cells, Peli1, ICOS, Infection, Autoimmunity, Lymphocyte differentiation

## Abstract

T follicular helper (Tfh) cells are crucial for regulating autoimmune inflammation and protective immunity against viral infection. However, the molecular mechanism controlling Tfh cell differentiation is poorly understood. Here, through two mixed bone marrow chimeric experiments, we identified Peli1, a T cell-enriched E3 ubiquitin ligase, as an intrinsic regulator that inhibits Tfh cell differentiation. *Peli1* deficiency significantly promoted c-Rel-mediated inducible T-cell costimulator (ICOS) expression, and *PELI1* mRNA expression was negatively associated with ICOS expression on human CD4^+^ T cells. Mechanistically, increased ICOS expression on *Peli1*-KO CD4^+^ T cells enhanced the activation of PI3K-AKT signaling and thus suppressed the expression of Klf2, a transcription factor that inhibits Tfh differentiation. Therefore, reconstitution of Klf2 abolished the differences in Tfh differentiation and germinal center reaction between WT and *Peli1*-KO cells. As a consequence, *Peli1*-deficient CD4^+^ T cells promoted lupus-like autoimmunity but protected against H1N1 influenza virus infection in mouse models. Collectively, our findings established Peli1 as a critical negative regulator of Tfh differentiation and indicated that targeting Peli1 may have beneficial therapeutic effects in Tfh-related autoimmunity or infectious diseases.

## Introduction

T follicular helper (Tfh) cells are a subset of CD4^+^ T cells that develop in B-cell follicles, where they interact with and assist B lymphocytes in germinal center (GC) formation.^1–3^ Upon interaction with B cells, Tfh cells promote the activation and induction of GC B cells, which is required for the terminal differentiation of these cells into long-lived plasma cells to produce antigen-specific, high-affinity antibodies.^2,3^ Therefore, Tfh cells play an essential role in protective immunity against infection by various pathogens, such as influenza virus. In addition, Tfh cells perform fundamental functions during the pathogenesis of antibody-mediated autoimmune diseases, such as systemic lupus erythematosus.^4,5^ Although the physiological function of Tfh cells has been extensively studied, the molecular mechanism controlling Tfh cell differentiation is poorly defined.

Peli1 is an E3 ubiquitin ligase that mediates both Lys63- and Lys48-linked polyubiquitination of specific protein substrates to modulate immune responses.^6–9^ We previously observed that compared to its homologous family members Peli2 and Peli3, Peli1 is highly and predominantly expressed in T lymphocytes,^10^ suggesting a potential nonredundant function of Peli1 in T-cell function. Indeed, we identified Peli1 as a pivotal negative regulator that inhibits T-cell activation and in vivo autoimmunity by modulating the K48-linked ubiquitination and degradation of c-Rel.^10^ More recently, a published study demonstrated that a microRNA, miR-155, is required for the generation and function of Tfh cells through targeting Peli1 and showed that heterozygous deletion of Peli1 in miR-155^−/−^ T cells substantially rescued impaired Tfh generation, suggesting a potential role of Peli1 in Tfh cells.^11^ However, whether and how Peli1 modulates Tfh cell differentiation and related biological functions remain undetermined. In the present study, we identified Peli1 as an intrinsic factor that suppresses Tfh cell differentiation through inhibition of inducible T-cell costimulator (ICOS) expression. As a consequence, deletion of Peli1 promotes Tfh-mediated antibody production in vivo, which promotes lupus-like disease but prevents influenza virus infection.

## Results

### Peli1 intrinsically inhibits Tfh differentiation

**Fig. 1:**
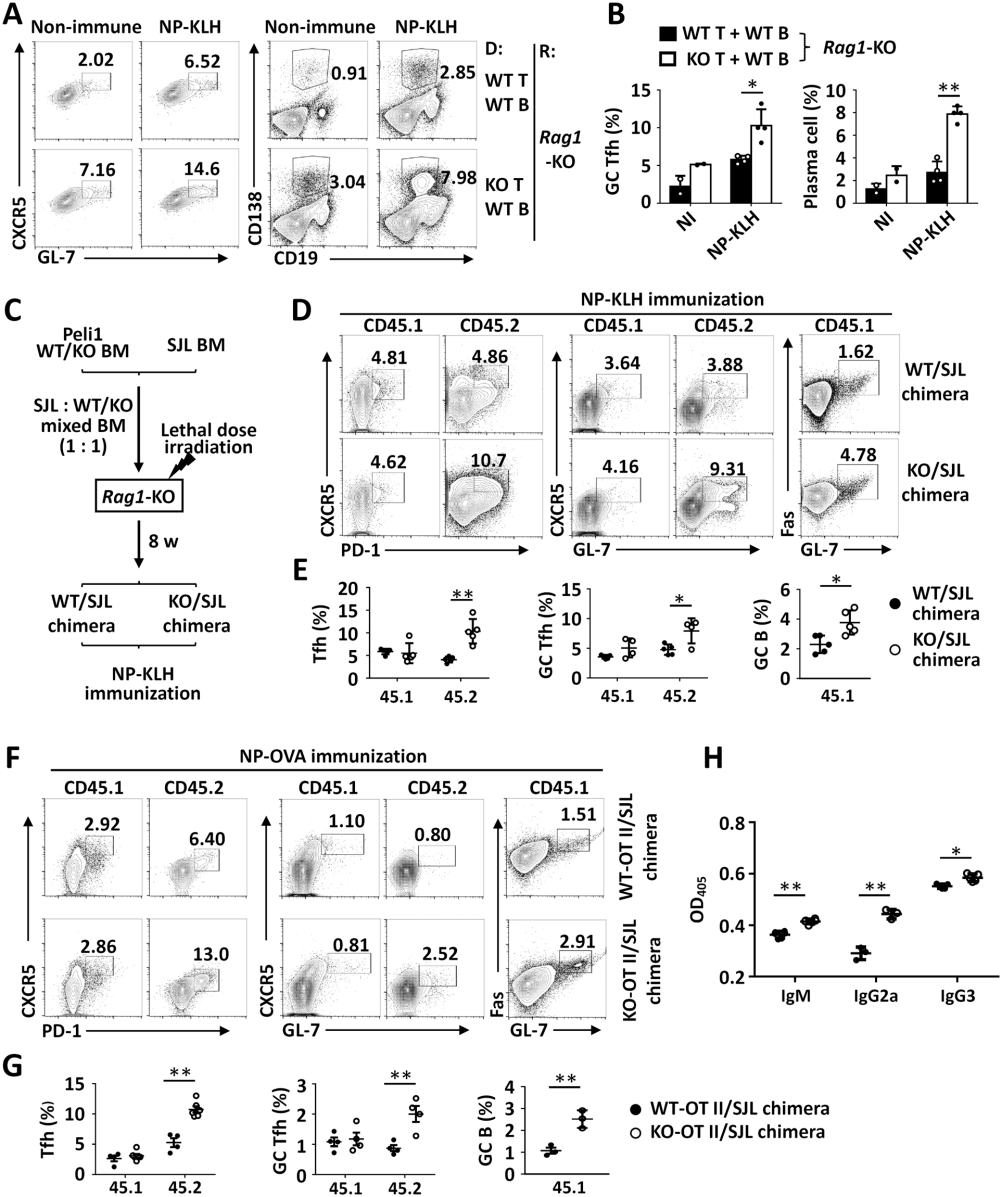
Peli1 intrinsically inhibits Tfh differentiation. **A**, **B** Flow cytometric analysis of the percentages of CXCR5^+^GL-7^+^ germinal center (GC) Tfh cells and CD138^+^CD19^−^ plasma cells in spleens from *Rag1*-deficient mice (recipient, R) that were adoptively transferred with WT or *Peli1*-KO T cells and WT B cells (donor, D) and were then immunized with or without NP-KLH. The data are presented as representative FACS plots (**A**) and summary graphs (**B**). **C** Scheme showing the generation of WT/SJL and *Peli1*-KO/SJL chimeric mice for NP-KLH immunization. **D**, **E** Flow cytometric analysis of the percentages of CXCR5^+^PD-1^+^ Tfh cells, CXCR5^+^GL-7^+^ GC Tfh cells and Fas^+^GL-7^+^ GC B cells in spleens from immunized WT/SJL and *Peli1*-KO/SJL chimeric mice as described in (**C**). The data are presented as representative FACS plots (**D**) and summary graphs (**E**). **F**, **G** Flow cytometric analysis of the percentages of CXCR5^+^PD-1^+^ Tfh cells, CXCR5^+^GL-7^+^ GC Tfh cells and Fas^+^GL-7^+^ GC B cells in spleen from NP-OVA immunized WT-OT II/SJL or *Peli1*-KO-OT II/SJL chimeric mice that generated similar to the scheme in (**C**). The data are presented as representative FACS plots (**F**) and summary graphs (**G**). **H** ELISA of NP-specific IgM, IgG2a, and IgG3 in the serum of NP-OVA-immunized WT-OT II/SJL and *Peli1*-KO-OT II/SJL chimeric mice as described in (**F**). Data with error bars are presented as the mean ± SEM values. Each panel shows data for a representative experiment from at least three independent biological replicates. ∗*p* < 0.05, ∗∗*p* < 0.01 as determined by unpaired Student’s *t* test

To investigate whether Peli1 regulates Tfh cell differentiation, we adoptively transferred WT or *Peli1*-deficient naive CD4^+^ T cells mixed with WT B cells into *Rag1*-knockout (KO) mice, which were then immunized with nitrophenol-keyhole limpet hemocyanin (NP-KLH) to induce the generation of Tfh cells in vivo. The results revealed that the loss of Peli1 significantly increased the frequencies of splenic GC Tfh cells upon NP-KLH immunization without affecting the differentiation of IFNγ-producing Th1 cells, IL-17A-producing Th17 cells and IL-4-producing Th2 cells (Fig. 1A, B; Supplementary Fig. 1). Accordingly, the increase in GC Tfh cells caused by *Peli1* deficiency dramatically promoted the generation of plasma cells (Fig. 1A, B).

To determine whether Peli1-mediated regulation of Tfh cell differentiation is T cell intrinsic, we generated mixed bone marrow (BM) chimeras in lethally irradiated *Rag1*-KO mice by reconstitution with a mixture of 50% BM from SJL mice and 50% BM from WT or *Peli1*-KO mice, and these chimeric mice were then immunized with NP-KLH 8 weeks post BM reconstitution (Fig. 1C). Therefore, both CD45.1^+^ SJL Tfh cells and CD45.2^+^ WT or *Peli1*-KO Tfh cells were generated in each immunized recipient chimeric mouse. Interestingly, we found that both groups of chimeras had similar percentages of splenic GC CD45.1^+^ Tfh cells. However, the KO/SJL chimeras showed a dramatic increase in the generation of splenic GC CD45.2^+^ Tfh cells, suggesting that Peli1 exhibits an intrinsic function of regulating Tfh differentiation. Accordingly, the induction of CD45.1^+^ GC B cells was also increased in immunized SJL/KO chimeric mice compared to WT/SJL chimeras (Fig. 1D, E).

Next, we generated another mixed BM chimera in *Rag1*-KO mice by reconstitution with a mixture of 50% BM from SJL mice and 50% BM from WT or *Peli1*-KO mice on the OT II background. In these chimeric mice, only WT-OT II BM- and *Peli1*-KO-OT II BM-derived T cells, not SJL BM-derived T cells, responded to NP-OVA immunization, which allowed us to examine antigen-specific induction of Tfh cells. As expected, we detected equal percentages of CD45.1^+^ Tfh and GC Tfh cells in WT-OT II/SJL and *Peli1*-KO-OT II/SJL chimeras after NP-OVA immunization. However, induction of CD45.2^+^ Tfh and GC Tfh cells was increased in *Peli1*-KO-OT II/SJL chimeras than in WT-OT II/SJL chimeras. Together with this induction, the generation of CD45.1^+^ GC B cells and production of serum antibodies were significantly increased in NP-OVA-immunized *Peli1*-KO-OT II/SJL chimeric mice compared to WT-OT II/SJL chimeras (Fig. 1F–H). Collectively, these results suggest that Peli1 functions as an intrinsic regulator to specifically inhibit Tfh cell differentiation in vivo.

### Peli1 deficiency promotes c-Rel-mediated ICOS expression

**Fig. 2:**
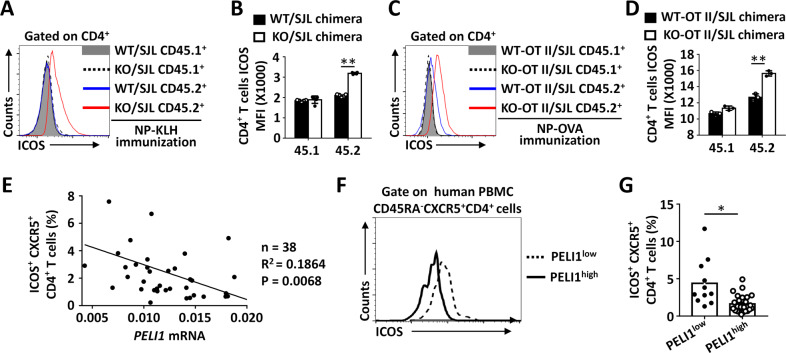
*Peli1* deficiency promotes ICOS expression. **A–D** Flow cytometric analysis of surface ICOS expression on CD4^+^ T cells isolated from NP-KLH-immunized WT/SJL and *Peli1*-KO/SJL chimeric mice (**A**, **B**) and from NP-OVA-immunized WT-OT II/SJL and *Peli1*-KO-OT II/SJL chimeric mice (**C**, **D**). The data are presented as representative FACS histograms (**A**, **C**) and summary graphs (**B**, **D**). **E** Correlation analysis of *PELI1* mRNA expression in PBMCs with the percentages of ICOS^+^CXCR5^+^CD4^+^ Tfh cells in human blood samples. **F**, **G** Flow cytometric analysis of surface ICOS expression on CD45RA^−^CXCR5^+^CD4^+^ T cells derived from PBMCs that expressed high (*PELI1*^high^) or low (*PELI1*^low^) levels of *PELI1* mRNA in human blood samples. The data are presented as representative FACS histograms (**F**) and summary graphs (**G**). Data with error bars are presented as the mean ± SEM values. Each panel shows data for a representative experiment from at least three independent biological replicates. ∗*p* < 0.05, ∗∗*p* < 0.01 as determined by unpaired Student’s *t* test

Published studies have suggested that ICOS is essential for Tfh differentiation, and deletion of ICOS sharply decreased the differentiation of Tfh cells and thus impaired GC formation and humoral responses in both human and mouse models.^12–16^ Interestingly, we found that surface ICOS expression was significantly increased on CD45.2^+^ *Peli1*-deficient CD4^+^ T cells isolated from KO/SJL or KO-OT II/SJL chimeric mice compared to that on CD45.2^+^ WT CD4^+^ T cells isolated from WT/SJL or WT-OT II/SJL chimeras after immunization with either NP-KLH or NP-OVA (Fig. 2A–D). In addition, *PELI1* mRNA expression was negatively correlated with surface ICOS expression on human Tfh cells, as reflected by the increased percentage of ICOS^+^CXCR5^+^CD45RA^−^ CD4^+^ T cells derived from PBMCs with low *PELI1* expression (Fig. 2E–G), suggesting that Peli1 is a negative regulator of ICOS expression in both mouse and human Tfh cells.

**Fig. 3:**
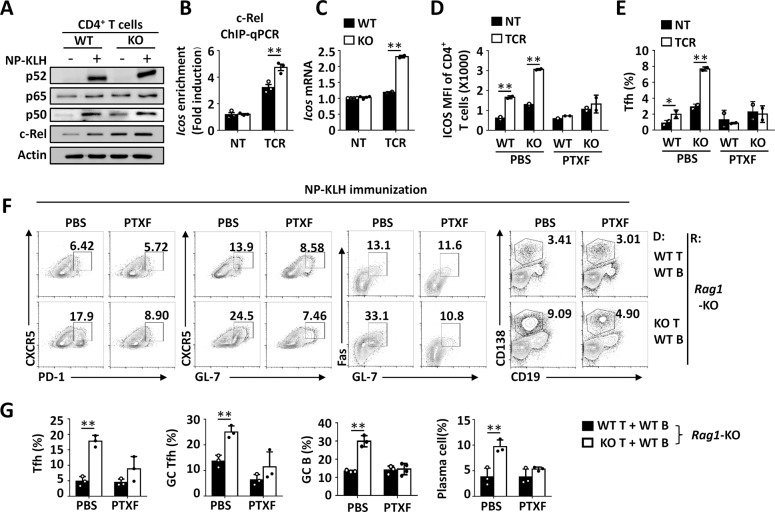
*Peli1* deficiency enhanced c-Rel-mediated ICOS expression. **A** Immunoblot of p52, p65, p50, c-Rel and actin (loading control) expression in splenic CD4^+^ T cells isolated from mice immunized with (+) or without (−) NP-KLH. ChIP-qPCR analysis of c-Rel binding activity at the *Icos* gene promoter (**B**) and qPCR analysis of *Icos* mRNA expression (**C**) in WT and *Peli1*-KO CD4^+^ T cells that were left untreated (NT) or treated with TCR stimulators (anti-CD3 plus anti-CD28 antibodies). **D**, **E** Flow cytometric analysis of ICOS expression on and Tfh differentiation of WT and *Peli1*-KO CD4^+^ T cells treated with PBS or pentoxifylline (PTXF) and then left untreated (NT) or treated with TCR stimulators (anti-CD3 plus anti-CD28 antibodies). **F**, **G** Flow cytometric analysis of the percentages of CXCR5^+^PD-1^+^ Tfh cells, CXCR5^+^GL-7^+^ GC Tfh cells, Fas^+^GL-7^+^ GC B cells and CD138^+^CD19^−^ plasma cells in spleens from *Rag1*-deficient mice (recipient, R) that were adoptively transferred with WT or *Peli1*-KO T cells and WT B cells (donor, D) and were then immunized with NP-KLH. The immunized mice were treated with PBS or PTXF daily on days 0–7 after immunization. The data are presented as representative FACS plots (**F**) and summary graphs (**G**). Data with error bars are presented as the mean ± SEM values. Each panel shows data for a representative experiment from at least three independent biological replicates. ∗*p* < 0.05, ∗∗*p* < 0.01 as determined by unpaired Student’s *t* test

We previously identified that the loss of Peli1 increased TCR-induced c-Rel protein expression in cultured CD4^+^ T cells,^10^ and a previous study suggested that c-Rel positively regulates Tfh cell differentiation.^11^ Therefore, we speculated that increased c-Rel levels in *Peli1*-KO CD4^+^ T cells may drive ICOS expression and finally contribute to enhanced Tfh cell differentiation. Similar to in vitro TCR stimulation, in vivo NP-KLH immunization specifically increased c-Rel protein levels in *Peli1*-deficient CD4^+^ T cells compared with WT cells (Fig. 3A). More interestingly, TCR stimulation induced robust binding of c-Rel to the promoter of the *Icos* gene, and *Peli1* deficiency further enhanced the DNA binding activity of c-Rel at the *Icos* gene promoter (Fig. 3B). Accordingly, Peli1 deletion dramatically promoted TCR-induced *Icos* mRNA expression in CD4^+^ T cells (Fig. 3C). In addition, in vitro TCR stimulation increased surface ICOS expression on and Tfh differentiation of *Peli1*-deficient CD4^+^ T cells compared to WT cells (Supplementary Fig. 2). To confirm that the *Peli1* deficiency-induced increases in ICOS expression and Tfh differentiation were due to increased protein levels of c-Rel, we used pentoxifylline (PTXF), a selective c-Rel inhibitor, to block c-Rel activity and then examined Peli1-mediated modulation of ICOS expression and Tfh differentiation. As expected, c-Rel inhibition dramatically suppressed ICOS expression and Tfh cell differentiation and abolished the difference between WT and *Peli1*-KO CD4^+^ T cells in the response to in vitro TCR stimulation (Fig. 3D, E). Moreover, c-Rel inhibition abolished the *Peli1* deficiency-induced enhancement of Tfh cell differentiation and GC formation upon in vivo NP-KLH immunization (Fig. 3F, G). Collectively, these data suggested that the increased c-Rel protein level in *Peli1*-deficient CD4^+^ T cells promoted ICOS expression and Tfh cell differentiation.

### Peli1 is required for Klf2 expression

**Fig. 4:**
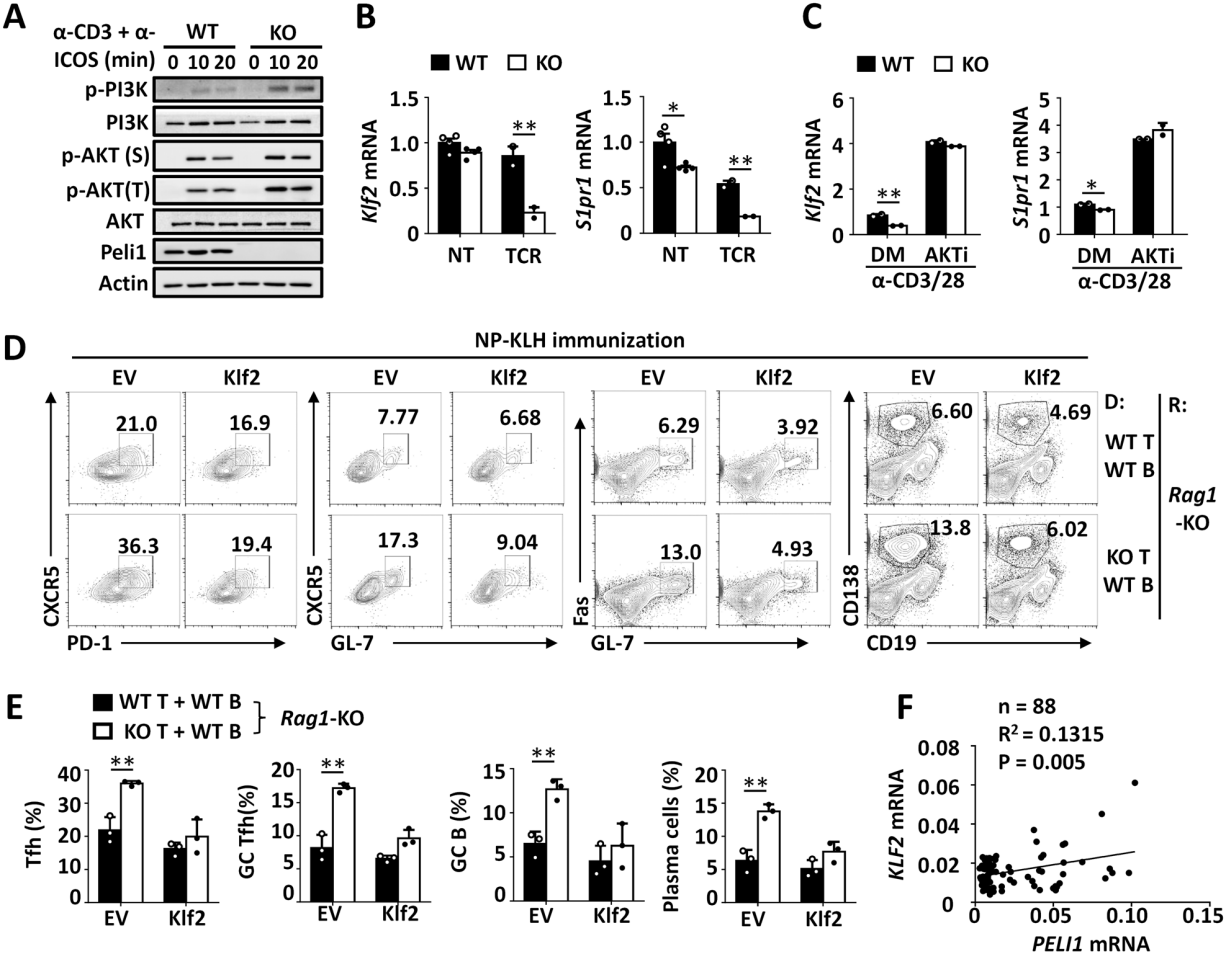
Peli1 is required for Klf2 expression to inhibit Tfh differentiation. **A** Immunoblot analysis of phosphorylated (p) and total PI3K and AKT, Peli1 and actin (loading control) in whole-cell lysates of WT and *Peli1*-deficient CD4^+^ T cells that were primed with anti-CD3 plus anti-CD28 antibodies for 24 h and were then left unstimulated or stimulated with anti-CD3 plus anti-ICOS antibodies at the indicated time points. **B**, **C** qPCR analysis of *Klf2* and *S1pr1* mRNA in WT and *Peli1*-deficient CD4^+^ T cells that were left untreated (NT) or treated with TCR stimulators (anti-CD3 plus anti-CD28 antibodies) (**B**) or were pretreated with DMSO (DM) or an AKT inhibitor (AKTi) and then stimulated with anti-CD3 plus anti-CD28 antibodies (**C**). **D**, **E** Flow cytometric analysis of the percentages of CXCR5^+^PD-1^+^ Tfh cells, CXCR5^+^GL-7^+^ GC Tfh cells, Fas^+^GL-7^+^ GC B cells and CD138^+^CD19^−^ plasma cells in spleens from *Rag1*-deficient mice (recipient, R) that were adoptively transferred with WT B cells plus WT or *Peli1*-KO T cells transduced with empty vector (EV) or retroviral vectors encoding Klf2 (donor, D) and were then immunized with NP-KLH. The data are presented as representative FACS plots (**D**) and summary graphs (**E**). **F** Correlation analysis of *PELI1* mRNA expression with *KLF2* mRNA expression in PBMCs from human blood samples. Data with error bars are presented as the mean ± SEM values. Each panel shows data for a representative experiment from at least three independent biological replicates. ∗*p* < 0.05, ∗∗*p* < 0.01 as determined by unpaired Student’s *t* test

ICOS ligation mediates the activation of downstream PI3K-AKT signaling.^17,18^ Since Peli1 is a negative regulator of TCR-induced ICOS expression, we speculated that Peli1 may negatively regulate downstream PI3K and AKT activation upon ICOS ligation. Consistent with this hypothesis, we confirmed that *Peli1* deficiency indeed enhanced the activation of PI3K and AKT upon combined anti-CD3 and anti-ICOS stimulation in TCR-primed CD4^+^ T cells (Fig. 4A). In addition, *Peli1* deficiency suppressed the expression of the transcription factors krüppel-like factor 2 (Klf2) and S1pr1, two downstream target genes of ICOS signaling (Fig. 4B). Moreover, AKT inhibition with a selective inhibitor dramatically increased the expression of Klf2 and S1pr1 and abolished the difference in mRNA expression levels between WT and *Peli1*-KO CD4^+^ T cells upon TCR stimulation (Fig. 4C).

A previous study suggested that ICOS-mediated Tfh cell maintenance is dependent on downregulation of Klf2,^19^ which prompted us to examine whether Peli1-mediated inhibition of Tfh cells is dependent on the maintenance of Klf2 expression. To this end, we overexpressed *Klf2* in WT and *Peli1*-KO CD4^+^ T cells, adoptively transferred these cells into *Rag1*-KO mice with WT B cells, and then immunized these mice with NP-KLH. As expected, Klf2 overexpression abolished the differences in the proportions of NP-KLH-induced Tfh cells, plasma cells and GC B cells within the WT and *Peli1*-KO CD4^+^ T-cell populations (Fig. 4D, E). In addition, the *Peli1* mRNA expression level was negatively correlated with the Klf2 mRNA level in human PBMCs (Fig. 4F). These results collectively suggest that Peli1 is required for the expression of Klf2, which maintains the suppression of Tfh cell differentiation.

**Fig. 5:**
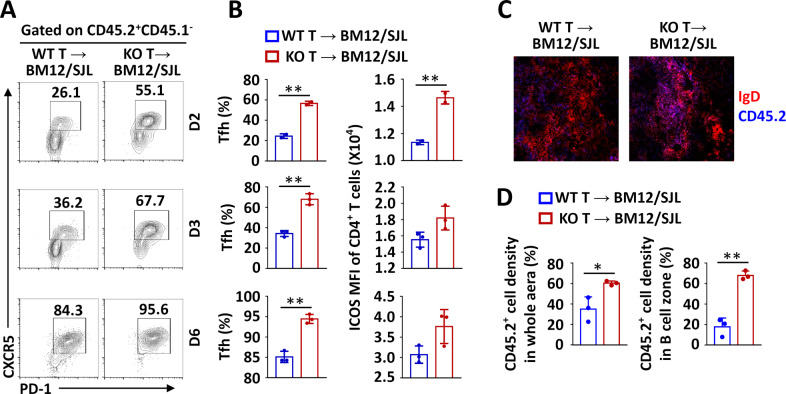
Peli1 deficiency does not affect Tfh cell migration. **A**, **B** Flow cytometric analysis of the percentages of CD45.2^+^CD45.1^−^CXCR5^+^PD-1^+^ Tfh cells (**A**) and ICOS expression in the spleens of bm12 and SJL mice immunized with CD45.2^+^ WT or *Peli1*-deficient CD4^+^ T cells for 2, 3, and 6 days. The data are presented as representative FACS plots (**A**) and summary graphs (**B**). **C** Immunohistochemical staining of draining lymph nodes from bm12 and SJL mice immunized with WT or *Peli1*-deficient CD4^+^ T cells. Cryosections of draining lymph nodes from immunized recipient mice were stained with CD45.2 (blue) and IgD (red) and analyzed by fluorescence microscopy. **D** Density of CD45.2^+^ cells in the whole area and the B-cell zone. Each dot represents an individual mouse. Data with error bars are presented as the mean ± SEM values. Each panel shows data for a representative experiment from at least three independent biological replicates. ∗*p* < 0.05, ∗∗*p* < 0.01 as determined by unpaired Student’s *t* test

ICOS-mediated PI3K-Akt signaling is required for Tfh cells to migrate from the T-B border to B-cell follicles.^20,21^ To examine whether Peli1 also modulates Tfh cell migration, we immunized bm12/SJL mice (bm12 mice under SJL background) by adoptive transfer of CD45.2^+^ WT or *Peli1*-deficient CD4^+^ T cells for Tfh induction.^7^ The results showed that the percentages of Tfh cells differentiated from *Peli1*-deficient CD4^+^ T cells were significantly increased on days 2, 3, and 6 compared to those of Tfh cells differentiated from WT CD4^+^ T cells (Fig. 5A, B). In addition, *Peli1* deficiency enhanced the expression of ICOS during the immunization period (Fig. 5B). We also examined the location of *Peli1*-deficient CD4^+^ T cells in draining lymph nodes during immune responses. Consistent with their increased frequency, the density of *Peli1*-deficient CD4^+^ T cells (identified by CD45.2 staining) in draining lymph nodes was much higher than that of WT CD4^+^ T cells (Fig. 5C, D). An increase in the *Peli1*-deficient CD4^+^ T-cell density was found in both B- and T-cell zones (Fig. 5C, D), suggesting that the migration of CD4^+^ cells from the T-cell zone into B-cell follicles is not affected by *Peli1* deficiency.

### Peli1 deficiency enhances Tfh cell-mediated biological functions

**Fig. 6:**
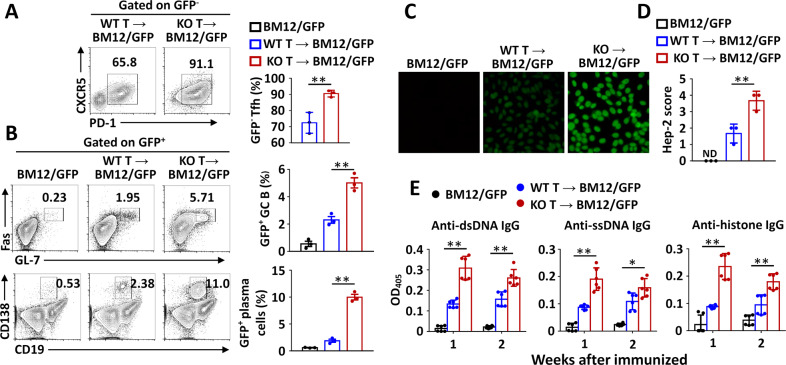
Peli1 deficiency promotes Tfh-mediated lupus-like pathology. **A**, **B** Flow cytometric analysis of the percentages of GFP^−^CXCR5^+^PD-1^+^ Tfh cells (**A**), GFP^+^Fas^+^GL-7^+^ germinal center (GC) B cells and GFP^+^CD138^+^CD19^−^ plasma cells in spleens of bm12/GFP mice adoptively transferred with WT or *Peli1*-deficient CD4^+^ T cells. The data are presented as representative FACS plots (left panels) and summary graphs (right panels). **C**, **D** Immunofluorescence analysis of serum anti-nuclear antibody (ANA) in bm12/GFP mice that were left unimmunized or transferred with WT or *Peli1*-deficient CD4^+^ T cells was performed with Hep-2 cells. The results are presented as immunofluorescence images (**C**) and a quantitative bar graph (**D**). **E** ELISA of anti-dsDNA, anti-ssDNA, and anti-histone IgG in the serum of bm12/GFP mice that were left unimmunized or adoptively transferred with WT or *Peli1*-deficient CD4^+^ T cells

To study the role of Peli1 in regulating Tfh-mediated physiological functions in vivo, we induced a lupus-like disease in bm12 mice (on the GFP background) by adoptive transfer of WT or *Peli1*-deficient CD4^+^ T cells. In this model, the donor-derived WT or *Peli1*-KO GFP^−^CD4^+^ T cells are recognized by recipient antigen-presenting cells and differentiate into Tfh cells, which promotes the expansion of recipient-derived GFP^+^ GC B cells and plasma cells and the production of autoantibodies.^7,8^ The results revealed that more donor-derived *Peli1*-KO GFP^−^CD4^+^ T cells than donor-derived WT cells were differentiated into Tfh cells (Fig. 6A). Together with Tfh induction, compared to transfer of WT cells, transfer of *Peli1*-KO CD4^+^ T cells induced robust increases in the populations of recipient GFP^+^ GC B cells and plasma cells (Fig. 6B). Accordingly, the production of serum anti-nuclear antibody, as reflected by Hep-2 scores (Fig. 6C, D), and the serum levels of anti-dsDNA, anti-ssDNA, and anti-histone IgG were significantly increased in *Peli1*-KO CD4^+^ T cell-immunized mice compared with WT cell-immunized mice (Fig. 6E). These data collectively suggested that increased Tfh differentiation of *Peli1*-deficient CD4^+^ T cells strongly promoted lupus-like autoimmunity.

**Fig. 7:**
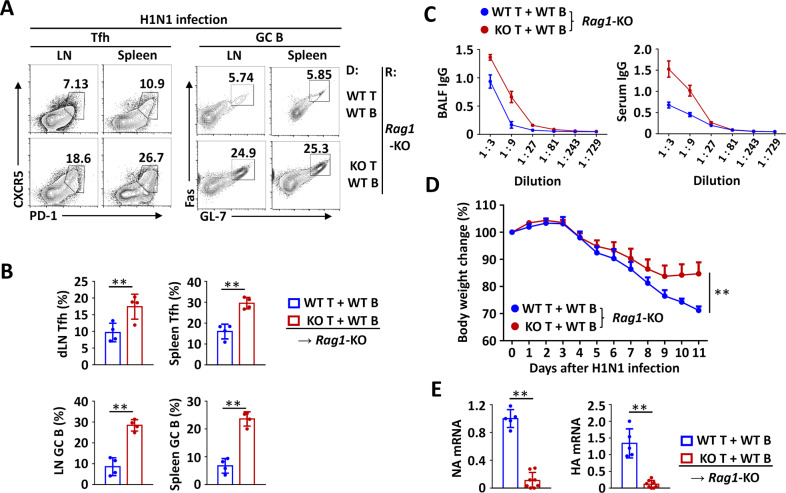
Peli1-deficient Tfh cells protect against H1N1 infection. **A**, **B** Flow cytometric analysis of the percentages of CXCR5^+^PD-1^+^ Tfh cells and Fas^+^ GL-7^+^ germinal center (GC) B cells in lymph nodes (LN) and spleens of *Rag1*-deficient mice (recipient, R) that were adoptively transferred with WT B cells plus WT or *Peli1*-KO T cells (donor, D) and were then infected with a lethal dose of the H1N1 Narita strain (2.5 LD_50_/mouse). The data are presented as representative FACS plots (**A**) and summary graphs (**B**). **C** ELISA of H1N1 virus-specific IgG in bronchoalveolar lavage fluid (BALF) and serum from infected mice. **D** The body weight change in *Rag1*-deficient mice (recipient, R) that were adoptively transferred with WT B cells plus WT or *Peli1*-KO T cells (donor, D) and were then infected with a lethal dose of the H1N1 Narita strain (2.5 LD_50_/mouse). **E** qPCR analysis of hemagglutinin (HA) and neuraminidase (NA) mRNA expression in the lungs of infected mice. Data with error bars are presented as the mean ± SEM values. Each panel shows data for a representative experiment from at least three independent biological replicates. ∗*p* < 0.05, ∗∗*p* < 0.01 as determined by unpaired Student’s *t* test

It is known that influenza virus infection induces the differentiation of host CD4^+^ T cells into Tfh cells, which in turn contribute to humoral immunity-mediated virus clearance.^22–25^ To examine the role of Peli1-modulated Tfh cells in regulating viral infection, we adoptively transferred WT or *Peli1*-deficient CD4^+^ T cells with WT B cells into *Rag1*-KO mice and then infected these mice with H1N1 influenza virus. Interestingly, we found that H1N1 infection-induced dramatic enhancements in the Tfh differentiation of transferred *Peli1*-KO CD4^+^ T cells compared to transferred WT cells and in the generation of GC B cells in both lymph nodes and spleens (Fig. 7A, B). Accordingly, the antibody levels in bronchoalveolar lavage fluid (BALF) and serum were also dramatically increased in mice that were transferred with *Peli1*-KO CD4^+^ T cells compared to those transferred with WT cells (Fig. 7C). Together with this trend, the increases of in vivo Tfh differentiation and antibody production significantly attenuated H1N1 infection-induced body weight loss in mice transferred with *Peli1*-KO CD4^+^ T cells compared to mice transferred with WT cells (Fig. 7D). In addition, the viral load was dramatically decreased in mice transferred with *Peli1*-KO CD4^+^ T cells, as reflected by the sharply decreased expression of both hemagglutinin (HA) and neuraminidase (NA), the two genes encoding the glycoproteins H1 and N1, in the lungs of virus-infected mice (Fig. 7E). Collectively, these results suggested that *Peli1* deficiency in T cells promoted Tfh-mediated humoral immunity and viral clearance.

## Discussion

Accumulating evidence has established the critical role of Tfh cells in initiating humoral immune responses and antibody production,^26–28^ which promote lupus-like autoimmunity but protect against viral infection.^4,5,23,29^ A previous study suggested that the microRNA miR-155 is critical for Tfh cell differentiation by potentially targeting Peli1,^11^ a T-cell-enriched E3 ubiquitin ligase.^10^ However, whether Peli1 can intrinsically modulate Tfh cell differentiation and Tfh-related physiological functions remains elusive. In the present study, through two mixed BM chimeric experiments, we found that Tfh cell differentiation was dramatically enhanced only for *Peli1*-deficient BM-derived CD4^+^ T cells, suggesting that Peli1 is an intrinsic negative regulator that inhibits Tfh cell differentiation. As a consequence, *Peli1* deficiency in T cells promoted lupus-like autoimmunity but protected against H1N1 influenza virus infection. Therefore, Peli1 is a potential novel molecular target for modulating Tfh cell differentiation, and targeting Peli1 may have a therapeutic effect on these Tfh cell-mediated pathological conditions.

ICOS is a costimulatory molecule that is essential for Tfh cell differentiation^12–16^ and has thus been recognized as one of the surface markers of Tfh cells.^5^ After interaction with the ICOS ligand, ICOS initiates the activation of the downstream PI3K-AKT signaling pathway.^17,18^ A recent study suggested that ICOS-mediated downstream signaling negatively regulates the expression of Klf2,^19^ a transcription factor that suppresses Tfh cell differentiation,^30^ and then contributes to the maintenance of Tfh cells in vivo. Our present study found that Peli1 suppressed ICOS expression through inhibition of c-Rel, which directly bound to the *Icos* gene promoter and mediated its transcription. Accordingly, Peli1 maintained the expression of Klf2 via suppression of ICOS expression and its downstream signaling, leading to inhibition of Tfh cell differentiation. Therefore, Peli1 is an upstream modulator that regulates ICOS-mediated Klf2 expression, which establishes a Peli1-ICOS-Klf2 axis controlling Tfh cell differentiation. Considering that the protein level of Peli1 is elevated in CD4^+^ T cells upon TCR stimulation, Peli1-mediated suppression of Tfh cell differentiation may constitute a negative feedback loop to inhibit overactivation of humoral immunity in vivo.

## Methods

### Human samples

Eighty-eight adult women were enrolled in this study, and blood samples collected from these women were used after informed consent was obtained. PBMCs were isolated from freshly collected blood and were then subjected to flow cytometry and real-time quantitative PCR analysis. Informed consent was obtained from all study subjects prior to their inclusion in this study. The sample collection protocol for this study was reviewed and approved by the Ethics Committee of Shanghai East Hospital, Tongji University, School of Medicine.

### Mice

Peli1^−/−^, Rag1^−/−^, and bm12 transgenic mice were used as previously described. SJL mice^7^ and OT II and GFP transgenic mice were purchased from Shanghai Model Organisms Center. In some experiments, bm12 transgenic mice were crossed with GFP transgenic mice or SJL mice to generate bm12 mice on the GFP or SJL background. All mice were maintained in a specific pathogen-free facility, and all animal experiments were performed in accordance with protocols approved by the institutional Biomedical Research Ethics Committee, Shanghai Institutes of Nutrition and Health, Chinese Academy of Sciences.

### Plasmid, antibodies, and reagents

MSCV-Klf2 was generated by the insertion of cDNA encoding full-length Klf2 into the MSCV-PIG (Puro-IRES-GFP) vector. Antibodies specific for Peli1 (sc-271065), c-Rel (sc-71), p52 (sc-7386), p50 (sc-1190), and p65 (sc-372) were purchased from Santa Cruz Biotechnology. Antibodies specific for p-PI3K (4228S), PI3K (4249S), p-AKT (Ser473, 4060S), and p-AKT (Thr308, 13038S) were purchased from Cell Signaling Technology. NP-KLH (N-5060) and NP-OVAL (N-5051) were purchased from Biosearch Technologies. PerCP-Cy5.5-conjugated anti-CD4 (45-0042-82), PE-Cy7-conjugated anti-ICOS (25-9942-80), PE-conjugated anti-CXCR5 (12-7185-80), APC-conjugated anti-PD-1 (17-9985-82), PerCP-Cy5.5-conjugated anti-CD19 (45-0193-82), PE-Cy7-conjugated anti-B220 (25-0452-82), PE-conjugated anti-CD138 (142504), PB-conjugated anti-GL-7 (48-5902-82), APC-conjugated anti-Fas (17-0951-82), FITC-conjugated anti-human CXCR5 (11-9185-41), APC-conjugated anti-human PD-1 (17-2799-41), and PE-Cy7-conjugated anti-human ICOS (25-9948-42) antibodies were purchased from eBioscience. V450-conjugated anti-human CD4 (560345) and PE-conjugated anti-human CD45RA (555489) were purchased from BD Biosciences. Live/Dead Fix Violet (L34963) was obtained from ThermoFisher. Mouse CD4 (L3T4, 130-049-201) and CD45R (B220, 130-049-501) MicroBeads were purchased from Miltenyi Biotec. InVivoMab anti-mouse CD3 (BE0001) and anti-mouse CD28 (BE0015) antibodies were purchased from BioXcell. Pentoxifylline (PTXF, P1784) was purchased from Sigma. The AKT inhibitor MK-2206 2HCl was purchased from Selleck. The c-Rel inhibitor pentoxifylline (PTXF, P1784) was purchased from Sigma.

### Cell culture

Plat-E cells were cultured in DMEM containing 10% FBS. Cells were seeded in six-well plates and transfected by the LipoFilter method. Primary naive CD4^+^ T cells were isolated from the spleen and lymph nodes with anti-CD4-conjugated magnetic beads (Miltenyi Biotec) and cultured in RPMI 1640 medium supplemented with 10% FBS. The isolated CD4^+^ T cells were left untreated or treated with anti-CD3 and anti-CD28 antibodies for the indicated durations. In some experiments, cells were treated with or without anti-CD3 and anti-CD28 antibodies along with the AKT inhibitor MK-2206 2HCl or the c-Rel inhibitor pentoxifylline. After treatment, cells were collected for further analysis by flow cytometry, immunoblotting or real-time quantitative PCR.

### Mouse immunization and antibody detection

Age- and sex-matched mice were immunized intraperitoneally with 200 µg of NP-KLH. After 0, 7, and 14 days, sera were collected and analyzed by enzyme-linked immunosorbent assay (ELISA). To establish the mouse model of lupus-like disease, 7.5 million purified WT or *Peli1*-KO naive CD4^+^ T cells were intraperitoneally injected into bm12/GFP recipient mice. Sera were collected at the indicated time points to evaluate anti-dsDNA, anti-ssDNA, and anti-histone antibodies by ELISA. Hep-2 cells were used for detection of serum anti-nuclear antibodies and were then visualized using a fluorescence microscope (ZEISS).

### Influenza virus infection

The influenza A virus (H1N1) strain A/PR8 was propagated in chicken embryos. To induce infection with influenza virus, mice were anesthetized with pentobarbital sodium and infected intranasally with mouse-adapted influenza virus strain A/Puerto Rico/8/34(PR8) (H1N1, Mount Sinai strain) at a dose of 450 TCID50 (half-maximal tissue culture infectious dose) per 30 μl. Then, the mice were weighed at the same time every day after infection.

### Adoptive transfer

To investigate the function of Peli1 in regulating Tfh cell differentiation, purified WT or *Peli1*-KO naive CD4^+^ T cells (1 × 10^6^ cells) mixed with WT B cells (1 × 10^6^ cells) were adoptively transferred into *Rag1*^−/−^ mice via intravenous injection. Twenty-four hours later, the mice were immunized intraperitoneally with 200 µg of NP-KLH or intranasally with H1N1 virus as described above, and then flow cytometric analysis of Tfh, GC Tfh, and GC B cells was conducted.

### BM chimeric mice

Lethally irradiated (^137^Cs, γ-ray, 950 rad) Rag1^−/−^ mice (8 weeks old) were adoptively transferred with mixed BM cells (1 × 10^7^ cells/mouse) isolated from SJL mice and WT (or WT-OT II) or *Peli1*-KO (or *Peli1*-KO-OT II) mice at a ratio of 1/1 via intravenous injection. After 8 weeks, the chimeric mice were immunized intraperitoneally with 200 µg of NP-KLH or NP-OVA as described above and were then used for flow cytometric analysis of Tfh, GC Tfh, and GC B cells and ELISA of antibody production.

### ChIP-qPCR

Chromatin immunoprecipitation (ChIP) assays were performed as previously described.^31^ Briefly, isolated WT and *Peli1*-KO naive CD4^+^ T cells (approximately 5 × 10^7^ cells) were fixed with 1% formaldehyde (Sigma-Aldrich) at room temperature for 10 min in 10 ml of medium prior to quenching with 125 mM glycine. Nuclear extracts were sonicated with a Covaris E220 sonicator for 660 s. After preclearing with normal IgG for 1 h, the sonicated cell lysates were immunoprecipitated with the anti-c-Rel antibody overnight on a nutator at 4 °C. The next day, protein A/G magnetic beads were added, and cell lysates were incubated on the nutator for another 2 h. After washing with buffers, chromatin was eluted from the protein/DNA complexes and digested with proteinase K and RNase A at 65 °C overnight to reverse crosslinking. The released DNA was purified with an AxyPrep PCR cleanup kit (Axygen) and subjected to quantitative PCR analysis with SYBR Green master mix. The sequences of the primers used for ChIP-QPCR are shown in Supplementary Table 1.

### Flow cytometry

Single-cell suspensions were prepared, stained with antibodies against different cell surface markers, and incubated on ice for 20 min; the cells were then resuspended in PBS with 2% FBS for flow cytometric analysis.^32^ The FSC/SSC gating strategy was initially applied, and antibodies conjugated to specific fluorochromes were then used to define the subsequent gates. All samples in the same experiments and comparisons were gated with the same parameters.

### Real-time qRT-PCR

Total RNA was isolated with TRIzol reagent (Invitrogen) and reverse transcribed to cDNA as previously described.^33^ RT-PCR was performed in triplicate with SYBR Green Supermix (Roche). The expression levels of individual genes were calculated by a standard curve method and normalized to the expression level of Actb. The gene-specific PCR primers are shown in Supplementary Table 1.

### Retroviral infection

For reconstitution of Klf2 in WT and *Peli1*-KO CD4^+^ T cells, MSCV-Klf2 was transfected into Plat-E cells with Lipofectamine 3000. After 48 h, retroviral supernatants containing fresh virus were collected for the infection of naive CD4^+^ T cells in the presence of plate-coated anti-CD3 plus anti-CD28 antibodies. The infected cells were selected by assessing GFP expression and sorted for adoptive transfer.

### Immunoblot

Immunoblot analysis was performed as previously described.^34^ *Rag1*^−/−^ mice were adoptively transferred with WT or *Peli1*-KO CD4^+^ T cells and WT B cells and were then immunized with NP-KLH. Seven days later, CD4^+^ T cells were collected from the immunized mice and subjected to immunoblot analysis. In some experiments, purified WT and *Peli1*-KO naive CD4^+^ T cells were pretreated with anti-CD3 plus anti-CD28 antibodies for 24 h, ‘rested’ overnight, and then stimulated with anti-CD3 and anti-ICOS (crosslinked with anti-hamster IgG) antibodies for the indicated durations. Whole-cell lysates were resolved using SDS-PAGE, transferred to nitrocellulose membranes (Millipore) and then subjected to immunoblotting with specific primary and secondary antibodies. Immunoblots were visualized using Immobilon Western Chemiluminescent HRP Substrate (Millipore) with a Luminescent Imaging Workstation (Tanon).

### Immunofluorescence microscopy

Mouse lymph nodes were washed with PBS, placed in optimal cutting temperature compound and snap frozen in dry ice. Cryosections with a thickness of 7 µm were fixed with 4% paraformaldehyde and were then stained with mouse IgD (BioLegend), goat anti-mouse Alexa Fluor 555 (Invitrogen), anti-CD45.2-Alexa Fluor^®^ 647 (BioLegend), and DAPI (Sangon Biotech). Images were acquired using a ZEISS cell observer and analyzed with ImageJ software.

### Quantification and statistical analysis

Statistical analyses were performed using GraphPad Software. Except where otherwise indicated, all presented data are representative results of at least three independent replicates. The data are presented as the mean ± SEM values, and *P* values were determined by two-tailed Student’s *t* tests. *P* values <0.05 were considered statistically significant.

## Supplementary information

Supplementary information
