# Supplementary material for: The ubiquitin ligase Peli1 inhibits ICOS and thereby Tfh-mediated immunity
Source: Cell Mol Immunol. 2021 Mar 11;18(4):969–78. doi: 10.1038/s41423-021-00660-5 (PMC8115645; doi:10.1038/s41423-021-00660-5)
Supplement: Supplementary file 1 — Supplementary information [file 41423_2021_660_MOESM1_ESM.pdf]

Supplementary Information

The ubiquitin ligase Peli1 inhibits ICOS and thereby Tfh-mediated immunity

by Huang *et al*.

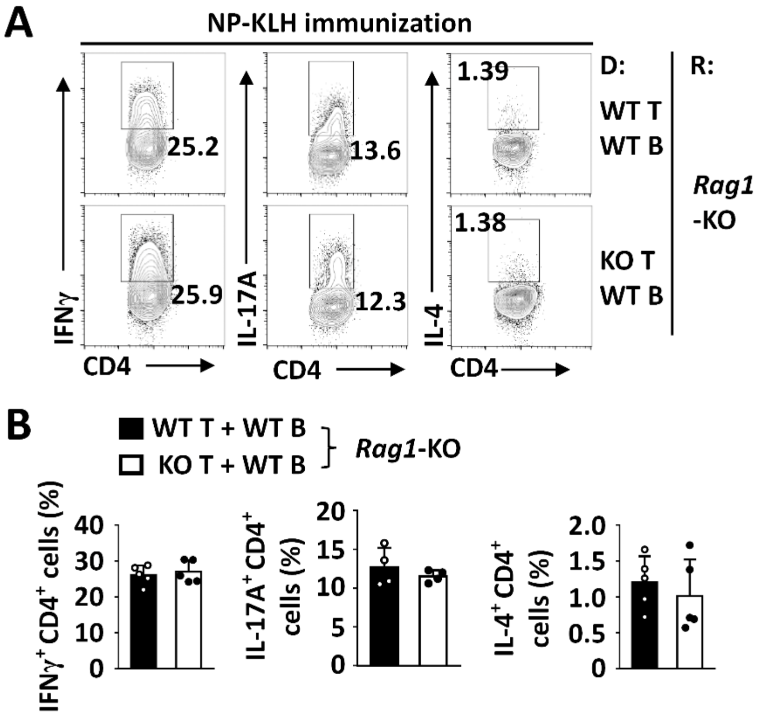

Supplementary Figure 1. Peli1 does not affect Th1/Th2/Th17 induction upon NP-KLH immunization

(A, B) Flow cytometric analysis of the percentages of IFNγ^{+}CD4^{+} Th1 cells, IL-17A^{+}CD4^{+} Th17 cells, IL-4^{+}CD4^{+} Th2 cells in spleen from *Rag1*-deficient mice (recipient, R) that were adoptively transferred with WT or *Peli1*-KO T cells plus WT B cells (donor, D), and then immunized with NP-KLH. Data are presented as the representative FACS plots (**A**) and summary graphs (**B**). Data with error bars are represented as mean ± SEM. Each panel is a representative experiment of at least three independent biological replicates.

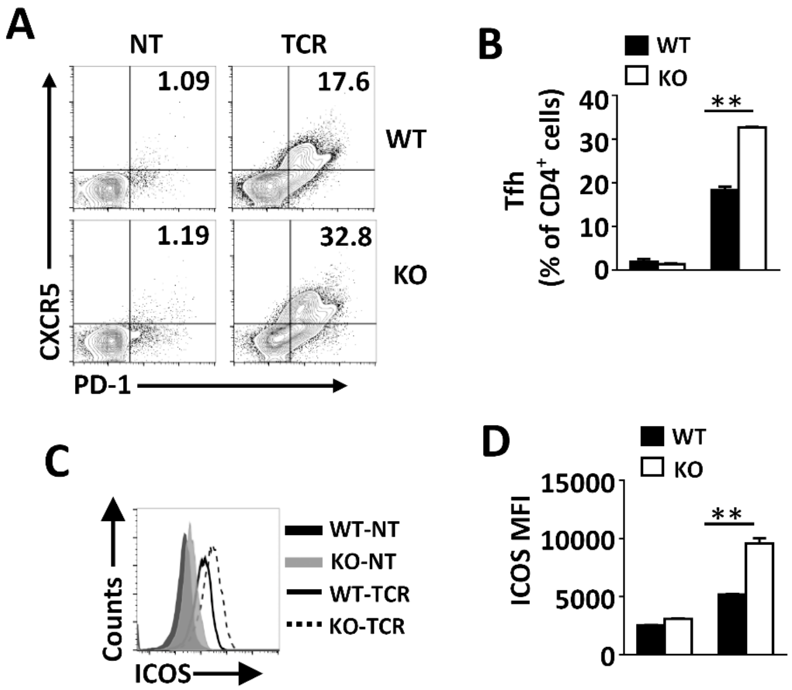

Supplementary Figure 2. *Peli1* deficiency promotes TCR-induced Tfh induction and ICOS expression

(**A-D**) Flow cytometric analysis of $CXCR5^{+}PD\text{-}1^{+}$ Tfh cells and ICOS expression in WT and *Peli1*-deficient $CD4^{+}$ T cells that were left nontreated (NT) or treated with TCR stimuli (anti-CD3 and anti-CD28). Data are presented as the representative FACS plots (**A**), histography (**C**) and summary graphs (**B, D**). Data with error bars are represented as mean ± SEM. Each panel is a representative experiment of at least three independent biological replicates. $*p < 0.05$, $**p < 0.01$ as determined by unpaired Student's t test.

Supplementary Table 1. Primers used for real-time quantitative PCR

**Genes**	**Forward Sequence（5'-3'）**	**Reverse Sequence（5'-3'）**
m*Actb*	CGTGAAAAGATGACCCAGATCA	CACAGCCTGGATGGCTACGT
m*Klf2*	GACCTACACCAAGAGCTCGC	CTTTCGGTAGTGGCGGGTAA
m*S1pr1*	GGCCACCACTTACAAGCTCA	TGTTGCTCCCGTTGTGTAGT
m*Icos*	TGACCCACCTCCTTTTCAAG	TTAGGGTCATGCACACTGGA
h*ACTB*	ACTCTTCCAGCCTTCCTTCC	CGTACAGGTCTTTGCGGATG
h*PELI1*	CGGCTCAGCAGAGAGGAAAA	TCACGGTAGGAGTGTGGGAA
h*KLF2*	GTCCTTCTCCACTTTCGCCA	ACAGGATGAAGTCCAGCACG
H1N1-HA	CTGCTCGAAGACAGCCACAA	CTGGGTTTCCCAAGAGCCAT
H1N1-NA	GGATCAATCTGTCTGGTAGTCGG	GTGTCCTTTACCCAGGTGCT
m*Icos* (ChIP)	AGTCACTTTAAACCTGACCCTATCA	GTCAGGGCTATTTACTCCAGAGA
